# Psychological distress prior to surgery is related to symptom burden and health status in lung cancer survivors

**DOI:** 10.1007/s00520-021-06537-7

**Published:** 2021-09-20

**Authors:** Marta Linares-Moya, Janet Rodríguez-Torres, Alejandro Heredia-Ciuró, María Granados-Santiago, Laura López-López, Florencio Quero-Valenzuela, Marie Carmen Valenza

**Affiliations:** 1 Department of Physical Therapy, Faculty of Health Sciences, University of Granada, Av. De la Ilustración, 60, 18016 Granada, Spain; 2 Hospital Virgen de Las Nieves, Granada, Spain

**Keywords:** Lung cancer, Health status, Survivors, Symptoms

## Abstract

**Purpose:**

Patients with lung cancer experience a variety of distressing symptoms which could adversely affect quality of life. The aim of this study was to determine whether psychological distress prior to surgery is associated to health status and symptom burden in lung cancer survivors.

**Methods:**

A longitudinal observational study with 1‐year follow‐up was carried out. Health status was measured by the WHO Disability Assessment Scale (WHO-DAS 2.0), the Euroqol-5 dimensions (EQ-5D) and the Pittsburgh Sleep Quality Index (PSQI). Symptoms severity included dyspnoea (Multidimensional Profile of Dyspnoea); pain (Brief Pain Inventory); fatigue (Fatigue Severity Scale); and cough (Leicester Cough Questionnaire).

**Results:**

One hundred seventy-four lung cancer patients were included. Patients in the group with psychological distress presented a worse self-perceived health status, functionality and sleep quality. The group with psychological distress also presented higher dyspnoea, fatigue and pain.

**Conclusion:**

Patients with psychological distress prior surgery present with a greater symptom burden and a poorer self-perceived health status, lower functionality and sleep quality, than patients without distress 1 year after the lung resection.

## Introduction

Lung cancer (LC) is the leading cause of cancer-related mortality worldwide [1], accounting for 27% of cancer deaths in 2014 [2]. Improvements in the diagnosis and treatment of LC have resulted in increased opportunities for long-term survival [3, 4]. Curative lung resection is the preferred treatment for early-stage lung cancer, significantly improving 5-year survival rates in this population [5]. This has led to a growing interest in addressing issues faced by these long-term survivors [6], identifying the physical and psychosocial factors affecting their well-being [7].

LC patients present various symptoms, such as pain, coughing, fatigue and shortness of breath in the early stages after surgery or even a long time after surgery [8–10]. Moreover, patients with lung cancer experience a variety of distressing symptoms, many of which begin before diagnosis and continue throughout the course of the disease and its treatments, adversely affecting functional status and quality of life (QOL) [11–13].

Psychological distress has been defined by the National Comprehensive Cancer Network as “an unpleasant experience of an emotional, psychological, social, or spiritual nature that interferes with the ability to cope with cancer treatment” [14]. It includes a wide continuum of psychological feelings relating to worry, anxiety, depression, fear and sadness and extends on a continuum from common normal feelings of vulnerability to problems that are disabling, such as true depression [15, 16].

Psychological distress prevalence rates in patients with cancer range from 22 to 58% [17, 18], with a higher prevalence among lung cancer patients when compared to patients with other cancers [19, 20]. Higher psychosocial distress can result from a late diagnosis, smoking, multiple symptoms, financial problems and health-related stigma [21–24], and could exacerbate disease-related symptoms contributing to poorer QOL outcomes well into survivorship [25]. This is consistent with theories such as the somatic perception hypothesis [26, 27] and somatosensory amplification [28], which posit that psychological factors, such as negative effects, can influence the perception and appraisal of somatic sensations or symptoms through their effects on higher cognitive processing patterns.

The clinical importance of assessing and treating psychological distress and mood disorder has received much attention from patient advocacy groups and health care providers, including consideration of distress as the sixth vital sign in cancer care [29]. However, despite being one of the most frequent cancers worldwide, little research has been done concerning the influence of psychological distress on symptom burden and health status in lung cancer survivors in the long term [30]. So, this study aimed to determine whether psychological distress prior to surgery is associated with health status and symptom burden in lung cancer survivors. We hypothesized that psychological distress before surgery could be related to a worse recovery in lung cancer survivors.

## Methods

A longitudinal observational study with a 1‐year follow‐up was carried out. We recruited patients above 18 years of age diagnosed with lung cancer and undergoing pulmonary resection, from the Thoracic Surgery Service of the “Virgen de las Nieves” Hospital Complex in Granada (Spain) between October 2018 and January 2020. All patients were informed about the purpose of the study and signed an informed consent form prior to their inclusion. Patients were excluded if they had one of these conditions: cognitive impairment or mental instability, physical disabilities that prevented their evaluation, inability to communicate, contraindication to physical exercise and presence of other respiratory pathologies. They were also excluded if they have suffered from any important pathology in the last year which could affect the study results. Ethical approval for this study was obtained from the Biomedical Research Ethics Committee of Granada.

Data collection was performed at admission and 1 year after hospitalization, by the same researchers, who had been previously trained. The evaluation was performed in the “Virgen de las Nieves Hospital Complex”, at admission, and in the Health Sciences Faculty of the University of Granada, 1 year after hospitalization.

Patients’ medical history was verified to confirm that they met the inclusion criteria. Data collected from the medical history included anthropometric data, comorbidities and lung resection characteristics, including type and duration of the surgery. Comorbidities were assessed by the Charlson index, one of the most widely used scoring systems for assessing comorbidities, and it has been validated in several disorders [31].

### Group assignment

Patients were divided into two groups based on the presence of psychological distress at hospital admission, assessed by the Hospital Anxiety and Depression Scale (HADS). The HADS is a questionnaire that has been previously used as a screening tool for psychological distress [32]. The questionnaire consists of 14 items, scored on a scale of 0–3. It is divided in turn into two subscales, one for anxiety (consisting of 7 items) and another for depression (consisting of 7 items), the higher score in each subscale greater anxiety or depression, respectively [33]. The cut-off point used was 11, based on previous studies with cancer patients [32].

### Outcome measures

Health status and symptoms severity were included as main outcomes. Health status was measured by the WHO Disability Assessment Scale (WHO-DAS 2.0), the Euroqol-5 dimensions (EQ-5D) and the Pittsburgh Sleep Quality Index (PSQI). Symptoms severity included dyspnoea, assessed by the Multidimensional Profile of Dyspnoea (MDP); pain, evaluated by the Brief Pain Inventory (BPI); fatigue, assessed by the Fatigue Severity Scale; and cough, measured with the Leicester Cough Questionnaire (LCQ).

WHO-DAS 2.0 is a generic tool for measuring health and disability in clinical practice, measuring changes in performance and their levels of difficulty in performing their activities. The scale consists of 36 items, divided into 6 domains, which are scored from 1 (slight) to 5 (extreme/unable to do so). The minimum score is 36 and the maximum is 180. This means that the greater the number, the greater the disability [34]. This test has high reliability and good validity [35, 36].

EQ‐5D is a validated tool to measure self‐perceived health status. It has been validated in Spanish [37]. It is divided into two sections, the first of which contains five items about mobility, self‐care, usual activities, pain/discomfort and anxiety/depression. These questions were scored between 1 and 3, where 1 represents “no problems” and 3 refers to “extreme problems”. The second section is a VAS that measures patients’ self‐evaluated health status from 0 to 100 (0 represents “the worst imaginable health” and 100 indicates “the best imaginable health”).

The PSQI is a self-rated questionnaire that assesses sleep quality and disturbances over a 1-month time interval [38], with strong reliability and validity [39]. It includes seven components: subjective sleep quality, sleep latency, sleep duration, habitual sleep efficiency, sleep disturbances, use of sleeping medication and daytime dysfunction.

The symptom severity assessment included dyspnoea, pain, fatigue and cough.

Dyspnoea was assessed with MDP that punctuates the general discomfort of the breath and the sensory and affective decreases of the dyspnoea [40, 41]. It also evaluates the dyspnoea at a specific moment or activity and it is valid and reliable for measuring these sensations in patients with respiratory problems [40]. It consists of 11 items, which the higher the score, the greater the dyspnoea perceived by the patient.

The pain was assessed with BPI, a questionnaire developed by Daut in 1983 and validated in Spanish by Badia et al. in cancer patients in 2002 [42]. BPI is a multidimensional pain assessment tool that provides information on the intensity of pain and its interference in patients’ daily activities. The version used includes 9 questions, the higher the score, the greater pain perceived by the patient [42], which has shown excellent reliability and validity in terms of psychometric evidence [43].

Fatigue was assessed with FSS, a self-administered questionnaire with 9 elements that assess the severity of fatigue in different situations [44]. The rating of each element varies from 1 to 7, where 1 indicates a strong disagreement and 7 strongly agree, and the final rating represents the average value of the 9 elements. The maximum score is the sum of all items, which would be 63, and the minimum 9. The higher the score, the more fatigue perceived by the patient (Valko PO et al., 2008). This scale showed good reliability and validity in terms of psychometric evidence.

Cough was assessed with LCQ [45], a questionnaire translated and validated into Spanish [46]. It is short and easy to administer, consisting of nineteen items with scores on a Likert scale ranging from 1 to 7. It is divided into three subscales: physical, psychological and social. The minimum and maximum score are 3 and 21, respectively, where a lower LCQ score means the presence of a higher cough.

### Statistical analysis

A priori power analysis with G*Power 3.1.9.2 software was performed based on a pilot study (unpublished) of fifteen subjects (effect size of 0.50) obtaining a statistical power of 95% and a sample size of 176 (88 per group). However, 97 participants per group were recruited to allow for a dropout rate of 10%.

Statistical Package SPSS version 20.0 (International Business Machines, Armonk, NY) was used to analyse the data obtained. Prior to statistical analysis, the Kolmogorov–Smirnov test was performed to assess the normality of the variables. Descriptive statistics (i.e. mean ± standard deviation) were carried out to describe sample baseline characteristics. Between-group comparison was performed after subjects were grouped by psychological distress, using the Student’s *t* test. Statistical significance was accepted at a *p* value of 0.05.

## Results

**Fig. 1:**
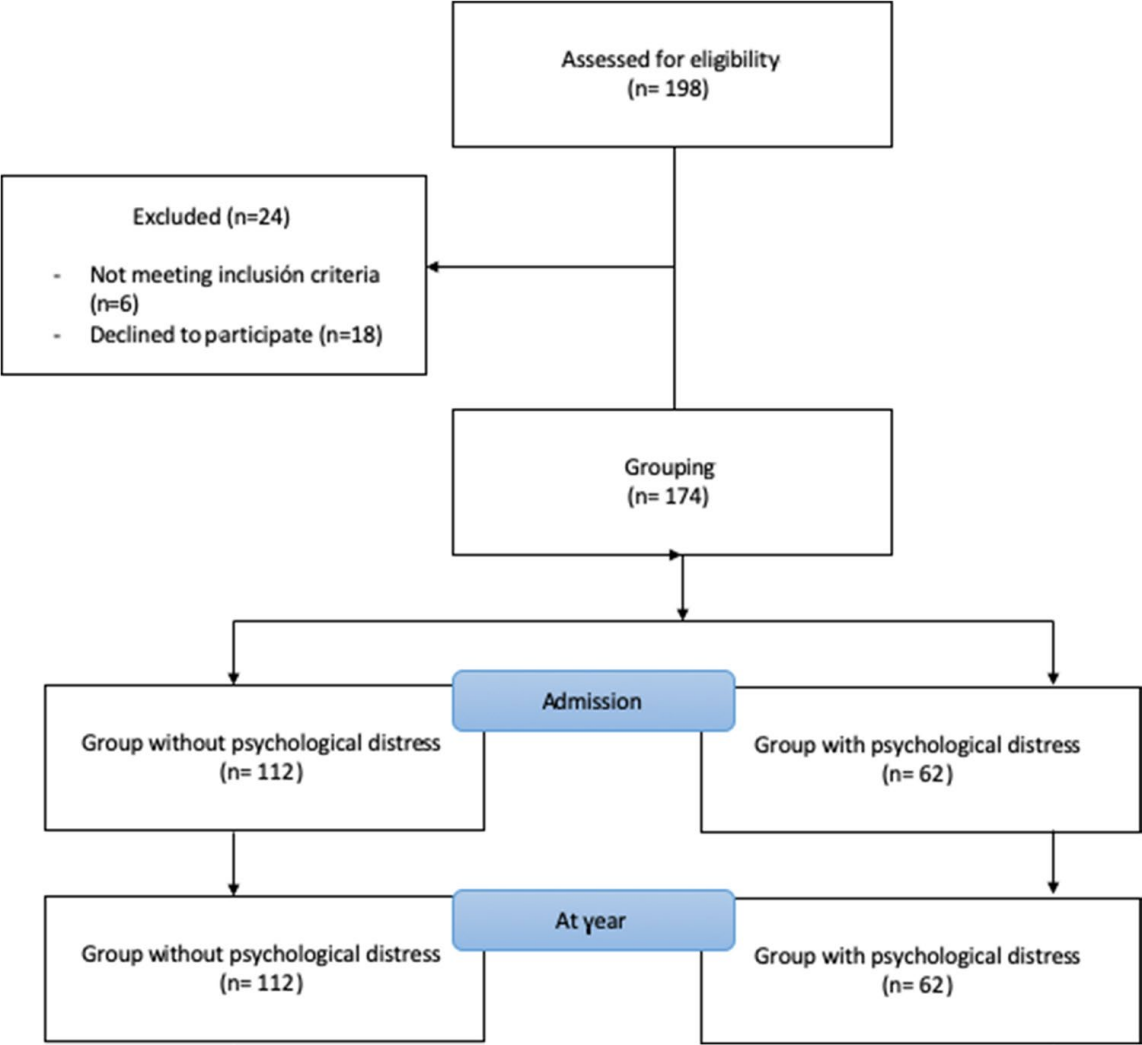
Flow diagram of participants

Of 198 potential patients, a final sample size of 174 was selected and divided into two groups depending on the presence of psychological distress. The distribution of patients is shown in Fig. 1. At first, 198 participants were recruited and, after checking the inclusion criteria and signed the informed consent, 24 participants were excluded. The HADS was used to divide the sample into two groups (112 vs 62).

Sociodemographic variables of the sample, duration of the intervention and length of hospital stay are presented in Table 1. The main descriptive variables of both groups are presented in this table.

**Table 1:** Sociodemographic variables of the sample, duration of the intervention and length of hospital stay

	Patients without psychological distress (*n* = 112)	Patients with psychological distress (*n* = 62)	*p*
Age (years)	56.60 ± 15.23	62.22 ± 11.11	0.095
Length of hospital stay (days)	6.68 ± 1.84	7.44 ± 2.08	0.102
Duration of intervention (minutes)	200.17 ± 71.53	203.89 ± 47.54	0.846
BMI (kg/${m}^{2}$)	27.12 ± 5.12	26.74 ± 3.60	0.732
MNA (total)	26.04 ± 3.11	25.73 ± 2.63	0.667
Sex (% men)	48.1	66	0.127
Charlson index	4.44 ± 2.42	4.37 ± 2.48	0.896

Data are expressed as mean ± SD or percentage (%);* BMI* body mass index;* SD* standard deviation; *FEV1%* forced expiratory volume in the first second in percentage; *p < 0.05, **p < 0.001

As we can see in Table 1, significant differences were found in sex, with a higher percentage of women in the group with psychological distress (*p* < 0.001). Nevertheless, no significant differences were found between groups in the other baseline characteristics such as age (*p* = 0.095), BMI (*p* = 0.732), comorbidities (*p* = 0.896) or MNA (*p* = 0.667). Length of hospital stay and duration of intervention were also similar in both groups; however, the group with psychological distress had a longer hospital stay and intervention.

The differences between preoperative and postoperative health status values, 1 year after hospitalization, are shown in Table 2. In this table, WHO-DAS 2.0, PSQI and ED-5D are reported, compared by group.

**Table 2:** Differences between preoperative and postoperative health status values, 1 year after hospitalization

	Patients without psychological distress (*n* = 112)	Patients with psychological distress (*n* = 62)	*p*
WHO-DAS 2.0			
WHO-DAS 2.0 cognition	7.55 ± 1.71	8.81 ± 4.39	0.005*
WHO-DAS 2.0 mobility	5.96 ± 1.63	6.25 ± 1.96	0.293
WHO-DAS 2.0 self-care	4.30 ± 0.62	4.41 ± 0.75	0.281
WHO-DAS 2.0 relations	5.75 ± 0.99	6.64 ± 2.26	< 0.001**
WHO-DAS 2.0 housework	5.36 ± 1.75	6.23 ± 2.34	0.006*
WHO-DAS 2.0 work and school activities	3.44 ± 3.73	3.96 ± 4.28	0.404
WHO-DAS 2.0 participation	10.09 ± 2.58	11.23 ± 4.32	0.031*
WHO-DAS 2.0 (total)	42.42 ± 7.39	47.58 ± 13.83	0.002*
EQ-5D			
EQ-5D VAS	80.35 ± 16.98	64.50 ± 20.57	0.002*
EQ-5D mobility	1.15 ± 0.36	1.30 ± 0.47	0.177
EQ-5D self-care	1.05 ± 0.22	1.30 ± 0.47	0.007*
EQ-5D usual activities	1.20 ± 0.40	1.40 ± 0.50	0.102
EQ-5D pain	1.10 ± 0.30	1.70 ± 0.47	< 0.001**
EQ-5D anxiety/depression	1.30 ± 0.46	1.80 ± 0.76	0.003*
Pittsburgh Sleep Quality Index			
Sleep disturbances	0.8 ± 0.39	1 ± 0	0.193
Use of sleeping medications	0.33 ± 0.78	1.2 ± 1.55	0.104
Daytime dysfunction	0.5 ± 0.80	0.4 ± 0.52	0.737
Subjective sleep quality	0 ± 0	0.6 ± 0.84	0.022*
Sleep latency	0.5 ± 0.52	0.4 ± 0.84	0.737
Sleep duration	0.5 ± 0.80	1 ± 1.33	0.289
Sleep efficiency	0.33 ± 0.78	1 ± 1.33	0.159
Total	3 ± 1.47	5.6 ± 3.8	0.041*

Data are expressed as mean ± SD or percentage (%);* WHO-DAS 2.0 *WHO Disability Assessment Scale; *EQ-5D* EuroQol-5D Health Questionnaire; *EQ-5D VAS *EuroQol-5D Visual Analogue Scale Health Questionnaire. *p < 0.05, **p < 0.001

Concerning to the self-perceived health status of the patients, 1 year after hospitalization, the group who presented psychological distress had worse scores in self-care (*p* = 0.007), pain (*p* < 0.001), anxiety/depression (*p* = 0.003) and VAS (*p* = 0.002).

The patients with psychological distress also presented worse scores in most of the WHO-DAS 2.0 domains (cognition (*p* = 0.005), relations (*p* < 0.001), housework (*p* = 0.006), participation (*p* = 0.031)) and the total score (*p* = 0.002).

Regarding the quality of sleep, the group with psychological distress had worse scores in most subscales, being statistically significant in subjective sleep quality (*p* = 0.022) and the total score (*p* = 0.041).

The differences between preoperative and postoperative symptoms, 1 year after hospitalization, are shown in Table 3. This table shows dyspnoea, pain, fatigue and cough differences.

**Table 3:** Differences between preoperative and postoperative symptoms, 1 year after hospitalization

	Patients without psychological distress (*n* = 112)	Patients with psychological distress (*n* = 62)	*p*
Multidimensional Dyspnoea Profile			
MDP E total	3.75 ± 5.58	17 ± 13,79	< 0.001**
MDP SQ	3.30 ± 6.48	11.30 ± 12.43	0.002*
Brief Pain Inventory			
Severity score	1.6 ± 4.63	10.60 ± 11.16	< 0.001**
Interference score	5.4 ± 13.26	7 ± 14.17	0.668
Total	7 ± 17.39	17.6 ± 21.08	0.043*
Fatigue Severity Scale	20.55 ± 13.98	30.40 ± 18.69	0.025*
Leicester Cough Questionnaire			
Physical	6.95 ± 0.1	6.17 ± 1.74	0.133
Psychological	7 ± 0	6.8 ± 0.42	0.114
Social	7 ± 0	7 ± 0	1
Total	20.95 ± 0.1	19.97 ± 2.16	0.129

Data are expressed as mean ± SD. *FSS* Fatigue Severity Scale; *MDP E* Multidimensional Profile of Dyspnoea Affective Scale;* MDP SQ* Multidimensional Profile of Dyspnoea Sensory Qualities Choice;* General MDP* Multidimensional Profile of General Dyspnoea;* BPI-SP* short questionnaire for the evaluation of pain; *LCQ* Leicester Cough Questionnaire; *p < 0.05, **p < 0.001

Statistically significant and clinical differences were found between groups in symptoms. The group with psychological distress presented higher dyspnoea (*p* < 0.001), fatigue (*p* = 0.025) and pain (*p* = 0.043) than the group without psychological distress, 1 year after hospitalization. The cough did not present differences between both groups, although the psychological distress group showed worse results.

## Discussion

This study aimed to determine whether psychological distress prior to surgery is associated with health status and symptom burden in lung cancer survivors in the long term. Our study shows that patients who present psychological distress before lung resection present poorer health status and more symptomatology 1 year after the intervention.

The sample of subjects included in this study was representative of the general population of patients with lung cancer, with a similar age range and surgery characteristics [47–49].

To the best of our knowledge, this is the first attempt to study psychological distress in LC survivors in the long term. Our results report that LC survivors who presented psychological distress prior to surgery present a greater symptom burden than patients without distress, with more dyspnoea, pain, fatigue and cough. This is supported by research, linking elevated psychological distress with poor physical, functional and psychosocial outcomes for patients with lung and other cancers [50–53]. Laird et al. [54] analysed a sample of 654 patients with advanced cancer and cachexia, reporting an important relationship between depression and the presence of pain and fatigue. In the same line, Fitzgerald et al. [55] analysed a group of 487 patients with cancer also reporting a significant relationship between depression, fatigue and pain. However, both studies did not focus on a specific type of cancer and they analysed the relationship between variables at a single time. Tishelman et al. [56] also noted that breathing, pain and fatigue were associated with the most distressed subjects in a sample of 400 patients with lung cancer. A recent study [23] also studied the presence of psychological distress in a sample of 2205 newly diagnosed patients with non-small lung cancer (NSLC). Their results showed that emotional problems were related to symptom burden, similar to our results; however, they did not follow the impact of psychological distress in the long term.

A decreased health status, in LC survivors who presented psychological distress prior to surgery, was found in our study, with poor functionality, self-perceived health status and sleep quality. Arrieta et al. [57] analysed a sample of 82 patients with NSLC and found an association between HADS score, quality of life and prognosis, even 6 months after treatment. However, they did not include the symptoms or other factors which could affect the quality of life. González-Saenz de Tejada et al. [58] carried out a similar study in patients with colorectal cancer. They explored the association of psychological status before surgery with changes in quality of life outcomes at 1-year post-intervention. Their results reported that patients with cancer, and absence of psychological distress, before surgery presented better results in quality of life outcomes at 1 year after surgery, similar to our study.

According to our results, PSQI scores have been associated with psychological distress. This corresponds with the literature where the patients’ levels of anxiety and depression have been associated with poor sleep quality [59, 60]. In the same line, Chang et al. [61] reported that the hypothalamic‐pituitary‐adrenal axis activation caused by increased psychological stress has a pronounced effect on the sleep quality of lung cancer patients. Our study also shows a reduced functionality 1 year after surgery in LC survivors with psychological distress. Similar results were found in the study of Lin et al. [51] where 145 lung cancer patients were analysed, and psychological distress was associated with lower functional status and worse quality of life. Cheville et al. [62] studied a sample of 2405 patients with lung cancer and showed similar results, relating psychological distress to functionality. These authors reported that psychological distress could even predict survival and functional status 5 years after diagnosis.

We should recognize potential limitations to this study. First, the severity of psychological distress was not evaluated over time, so the temporal relationship between change in these problems, quality of life and symptom burden is unknown. However, our study design has based on previous studies where psychological distress was only evaluated once [32, 60]. Second, the lack of a structured psychiatric clinical interview to assess depression and anxiety is also one of the limitations. Nevertheless, previous studies have used the HADS to evaluate the presence of psychological distress [11, 63].

Our findings demonstrate that psychological distress is an important consideration in the care of patients with lung cancer and that a brief screening of these problems prior to surgery can predict the evolution of symptoms and health status in the long term. A better understanding of the impact of psychological distress on cancer survivors could raise awareness, promote the development of better treatment strategies and improve the quality of life of these patients. So, future studies developing interventions that approach these disorders may be useful to improve the recovery and prognosis of these patients.

Therefore, the clinical impact of our results is evident. Detecting patients with psychological distress prior to surgery should be included in the usual clinical practice, due to its relevance. Clinicians, psychologists or nurses, among others, should know prognosis factors that could affect the lung cancer population.

## Conclusion

Psychological distress is an important factor to take into account in lung cancer survivors. Patients with psychological distress prior to surgery present a greater symptom burden, with more dyspnoea, cough, fatigue and pain. With regard to health status, LC survivors with psychological distress before surgery presented a poorer self-perceived health status and lower functionality and sleep quality, than patients without distress 1 year after the lung resection.

## Data Availability

Not applicable.
